# Supplementary material for: Forecasting the spread of COVID-19 under different reopening strategies
Source: Sci Rep. 2020 Nov 23;10:20367. doi: 10.1038/s41598-020-77292-8 (PMC7683602; doi:10.1038/s41598-020-77292-8)
Supplement: Supplementary file 1 — Supplementary information. [file 41598_2020_77292_MOESM1_ESM.pdf]

Technical Appendix: Forecasting the Spread of COVID-19 under Different Reopening Strategies*

Meng Liu Raphael Thomadsen Song Yao

Olin Business School
Washington University in St. Louis

This draft: August 10, 2020

*Please contact Liu (mengl@wustl.edu), Thomadsen (thomadsen@wustl.edu), or Yao (songyao@wustl.edu) for correspondence.

In this online appendix, we first present our data. We then discuss the sensitivity of our results to the assumed contagious period. Next, we provide some supplemental details to our simulations. Finally, we lay out how the concavity we estimate could come from a model of interconnected networks.

A Data

Our data come from a multitude of sources. We lay out the sources for each of these in turn.

A.1 Positive Cases

Data of positive cases are based on the COVID-19 data published by the New York Times (`https://github.com/nytimes/covid-19-data`, accessed on August 6, 2020). The data contain the daily confirmed case counts for 2,953 U.S. counties or county-equivalents. The case data for the five boroughs of New York City, however, are not recorded separately by New York Times. In this case, we use the data published by the Health Department of New York City in lieu of the five boroughs (`https://github.com/nychealth/coronavirus-data`, accessed on August 6, 2020). The case data of Kansas City, Missouri are also recorded separately because it overlaps with 4 adjacent counties. We attribute the cases of Kansas City to Jackson County, Missouri because most of the city lies within Jackson County. We also drop 3 counties because we do not have social distancing data for 2 of them, and we cannot match the population data for a third (Oglala Lakota County, SD). Finally, we remove counties that had no confirmed cases during our estimation sample period of Feb 1, 2020 to May 23, 2020. After all the above-mentioned filters, we have a panel of 2,924 counties. These counties account for 99.76% of the US population and 99.91% of the total U.S. confirmed COVID-19 cases till August 6, 2020.

There are a few days where there are negative cases that are reported. These are generally corrections to previous over-reporting. Thus, we clean the negative numbers of cases by subtracting the absolute value of the negative cases from the proceeding day. In the event that that leads to a negative number of the proceeding day, we iterate again.

A.2 Social Distancing

We use social distancing data from the company SafeGraph, which collects cellphone GPS data from U.S. residents, and has made them available for free to academics studying COVID-19. These data are collected through a series of pings that the company receives for all users who have installed a number of smartphone apps. The list of apps that collect this information is kept as a trade secret. We measure social distancing as the first principle component of several measures. The measures are, on a given day, percentage of residents staying home, percentage of residents working at workplace full-time, percentage of residents working at workplace part-time, median duration of residents staying home, and median distance of residents traveled. The SafeGraph data are published at the Census Block Group level. To accommodate other data sources which are

available at a less granular level, we aggregate the this variable to the county level by taking the weighted median, using the number of cellphones in each Census Block Group as the weight.

A.3 Demographic data

We obtain the demographic data from the Census Bureau's 2014-2018 American Community Survey (ACS), which contains information of each county's profile of population, ethnicity, age, median income, and commuting pattern. The ACS, however, does not report population densities. SafeGraph, the company who provides us with the social distancing data, also maintains a dataset of the land area of each Census Block Group in the US. We aggregate the land areas to the county level. Together with the county population information from the Census Bureau, we are able to construct the population density data of each county.

A.4 Weather data

We gathered historical daily rain and temperature data from National Oceanic and Atmospheric Administration (NOAA) (source: `https://www.ncei.noaa.gov/metadata/geoportal/rest/metadata/item/gov.noaa.ncdc:C00861/html`, accessed on May 21, 2020). The raw weather data is at the weather station level and we match weather stations to the counties they are in. We use the average values across weather stations within the same county to construct the weather variables for that county. For a small number of counties where there are no associated weather stations, we use the daily state averages as proxies.

A.5 Putting it all together

Our sample is an unbalanced panel because counties start to have positive number of confirmed cases on different dates. The earliest date we observe in the sample is Jan 29, 2020, and the last day is August 6, 2020. Note that we construct actual cases using reported cases 5 days later, and thus the corresponding sample period based on reported cases is Feb 3, 2020 to August 1, 2020.

Summary statistics of all of the variables we use in the estimation are presented in Table A1. Note that our humidity data end on May 18, 2020. For estimation we only use case data up to May 18. But our case data proceed past the dates used for estimating the model and we use those data for validating the model. Those data are publicly available, and we have also posted the compiled case data and the code for compiling the data at `https://github.com/songyao21/covid_data_depot`.

B Sensitivity to Duration of Contagious Period

Research on COVID-19 is nascent, and there are different views of how long infected individuals stay contagious. Suppose that such individuals are contagious for 14 days instead of 6 days. Then the model becomes:

$$y_{i,t} = R_{i,t}S_{i,t}\left(Y_{i,t-2} - Y_{i,t-16}\right)^{\omega}. \tag{A1}$$

Time-varying Variables	(1) N	(2) Mean	(3) Std Dev.	(4) Min	(5) Max
Reported cumulative cases	131,272	337.4	2,178	0	63,690
Reported new daily cases	131,272	11.40	64.40	0	2,174
Average temp (in Celsius)	131,272	12.65	6.733	-16.40	32.06
Rain (mm)	131,272	3.469	7.937	0	145.1
Humidity (percentage)	131,272	66.95	15.24	8.745	100
Social_distancing	131,272	1.115	1.029	-4.578	4.878
Time-invariant Variables					
Median income ($)	2,923	52,580	14,606	19,391	144,821
Population	2,924	111,906	344,510	625	1.004e+07
Population density (people / square miles)	2,924	292.5	1,860	0.0359	71,891
Share of population senior (>=70)	2,924	0.122	0.0327	0.0229	0.384
Share of population youth (<=17)	2,924	0.224	0.0338	0.0732	0.403
Share of population black	2,924	0.096	0.148	0	0.874
Share of population Hispanic	2,924	0.092	0.135	0	0.991
Share of population public transit commuters	2,923	0.010	0.0333	0	0.642

Table A1: **Summary Statistics**

We present the estimation results of this model in column 2 of Table A2. Note that this regression has more observations because there are fewer instances where we observe no cases in a county for a 14-day window than for a 6-day window. The results are largely unchanged. The coefficient on social distancing levels are slightly lower, but well within one standard error of the corresponding coefficient in column 1. The exponent on the infectious individuals is 0.523. That is somewhat smaller (but statistically different) than the 0.571 we observe with the shorter 6-day window, but overall the curvature shape is similar to what we have observed with the 6-day window. As we will discussion below in Section C, both specifications give similar long-run forecasting results.

We next regress the county fixed effects on several demographic variables, which are reported in Table A3. Comparing the results for the 6 day vs. 14 day contagious periods, we see that over all the results are qualitatively very similar. The main difference is that if the contagious period is 14 days we observe that children are also statistically more contagious than non-senior adults.

C Simulation

We forecast the cumulative and daily cases of COVID-19 through the end of October at different levels of social distancing. Those forecasts appear in Figure 2 in the original paper. In Figure A1 below, we replicate the graph with cumulative cases, but further add confidence intervals. To avoid cluttering, we only depict current and 75% return-to-normalcy levels in A1. We observe that these forecasts are indeed statistically significantly different.

As a robustness check regarding the 6-day contagion window specification, we also consider forecasting US daily cases under the specification where the contagion window is 14 days. Figure A2 shows the evolution of daily cases till October 31, 2020 under current-level (early August) and

	(1) Contagious for 6 days	(2) Contagious for 14 days
Dependent Variable	Log(Infected in County i on Date t)	Log(Infected in County i on Date t)
Social Dist. Level in County i on Date t	-0.824***	-0.725***
	(0.245)	(0.215)
Infectious Individuals in County i on Date t	0.571***	0.523***
	(0.014)	(0.014)
Avg. Temperature ($°C$) of County i on Date t	-0.001	0.002
	(0.002)	(0.002)
Avg. Humidity of County i on Date t	0.005**	0.004**
	(0.002)	(0.002)
County Fixed Effects	Yes	Yes
Date Fixed Effects	Yes	Yes
Observations	131,272	148,946
R_squared	0.63	0.64
Counties	2,924	2,924

*** p<0.01, ** p<0.05, * p<0.1

Table A2: **Estimation of a Modified SIR Model.**

75% return-to-normalcy regimes. We overlay the forecasts of both 14-day and 6-day specifications for easy comparison. From the figure, we may see the forecasts of 14-day and 6-day contagion window specifications are fairly close.

D Concavity of SIR model and Network Dynamics

A unique feature of our model is that we estimate an exponent on the number of contagious cases. We include this flexibility because such a model fits the data much better, and also leads to forecasts that have more limited growth after an initial take-off of COVID-19 cases, as is commonly observed. We illustrate that the concave relationship we estimate for the number of contagious individuals on the number of new cases can come from social networks between people through a very simplified model of networks and disease process.

To do this, we simulate a network with the following process: We take 10,000 individuals. We create a network by first randomly assigning that any two individuals will be joined with a common node with probability 11/20,000. Call these connections "round-1 friends." We then expand this network by assigning each node to have an edge with each of the friends of round-1 friends with a probability of 0.8.

We assume that the disease spreads with the following process. We seed 4 individuals to have the disease in period 0. Then in each period we assume that any connected individual will get sick with probability 0.4.

After simulating this process, we then regress $(\ln(y_t) - \ln(S_t)) = c + \omega \ln(y_{t-1}) + \varepsilon_t$. The mean value for $\hat{\omega} = 0.57$. This shows the plausibility of network effects leading to an estimate in the

	(1) Contagious for 6 days	(2) Contagious for 14 days
Dependent Variable	County Fixed Effect	County Fixed Effect
Log(Pop. Density)	0.4410***	0.4006***
(People/Sq. Miles)	(0.0096)	(0.0095)
Fraction Black Residents	0.8084***	0.7658***
	(0.0979)	(0.0970)
Percentage Hispanic	1.3438***	1.3112***
Residents	(0.1003)	(0.0993)
Percentage of Commuters	5.0215***	5.5818***
using Pub. Transportation	(0.4140)	(0.4101)
Log(Median Income)	1.3387***	1.2564***
(in U.S. dollars)	(0.0579)	(0.0573)
Percentage of Senior	1.8825***	2.2584***
Residents (≥70yrs)	(0.5255)	(0.5205)
Percentage of Children	0.6614	1.0609**
Residents (<18yrs)	(0.4733)	(0.4688)
Constant	-16.6781***	-15.7657***
	(0.6462)	(0.6401)
R_squared	0.69	0.66
Counties	2,923	2,923

*** p<0.01, ** p<0.05, * p<0.1

Table A3: **Analysis of County Fixed Effects**

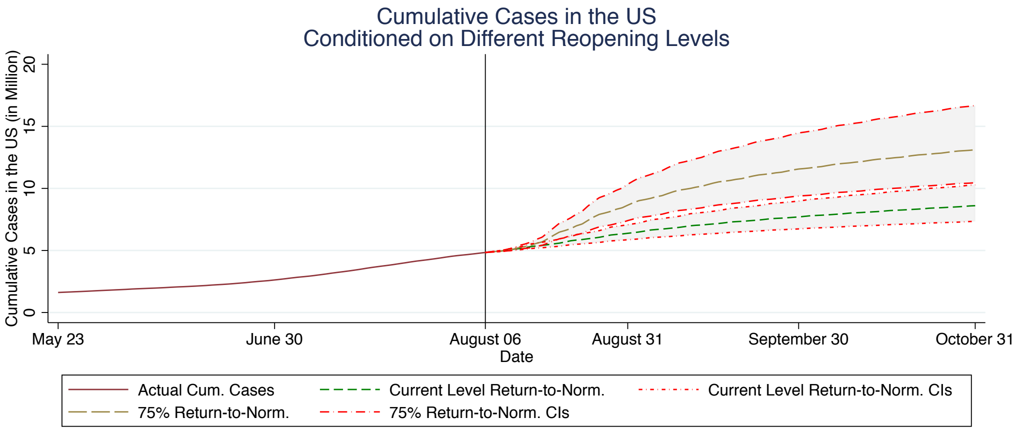

Figure A1: **Cumulative Case Forecasting under Different Reopening Strategies with Confidence Intervals.** The vertical line indicates the last day of diagnosed case data sample.

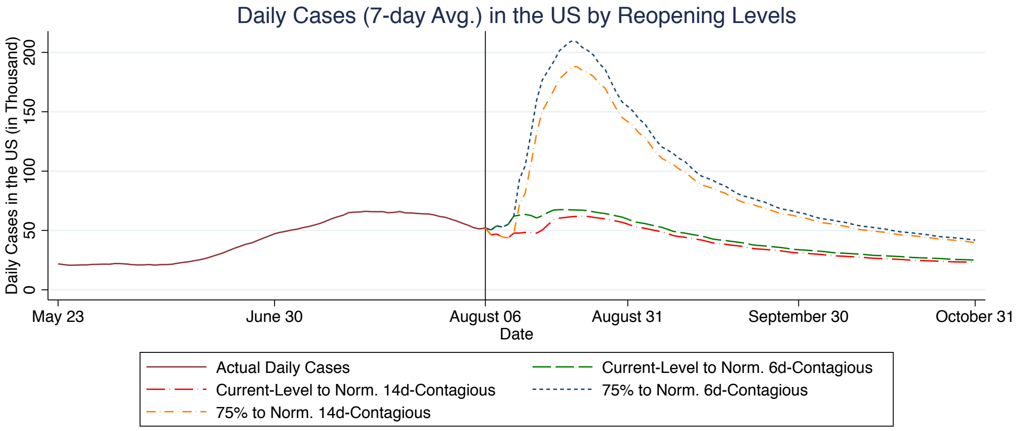

Figure A2: **Daily Case Forecasting under Different Contagion Window Specifications.** The vertical line indicates the last day of diagnosed case data sample.

range that we have estimated in our main model. We have placed the R code for this simulation at `https://github.com/songyao21/covid_data_depot/network_simulation`, so that interested readers can play with the parameters to understand the process more.
